# Setting up the CoLaus study

**DOI:** 10.1186/1753-6561-7-S5-O6

**Published:** 2013-08-30

**Authors:** P Vollenweider

**Affiliations:** 1 University Hospital of Lausanne, Switzerland

## 

The CoLaus study is a large population based cross sectional study in over 6000 subjects aged 35-75 years living in Lausanne, Switzerland. Its main goals are to obtain information on (1)The prevalence of cardiovascular risk factors and diseases in the population of Lausanne, (2)New biologic and genetic determinants of isolated and clusters of cardiovascular risk factors.

Recruitment was done from 2003 to 2006 to obtain a representative sample of subjects. The participation rate was 42%. The participants were extensively phenotyped by administering a questionnaire on personal and family history of cardiovascular risk factors & personal medical history, a basic physical exam for cardiovascular risk factors, mental status examination and biological markers in blood and urine. Plasma, serum, whole blood and urine were stored at -80^o^C and all Caucasian subjects were genotyped with database in Lausanne.

Starting in 2004, over 3600 CoLaus participants underwent an extensive psychiatric phenotypization (PsyCoLaus). This extensive phenotype should allow us to better understand the known association between cardiovascular diseases and mental health.

In May 2009 follow-up of the entire CoLaus population was started to prospectively assess the association between cardiovascular diseases, cardiovascular risk factors and mental disorders. Data were collected to know the course of the conditions related to cardiovascular diseases or mental disorders observed at baseline, the incidence of such conditions during the follow-up and the evaluation of candidate variables which could be either mediators of a causal relationship or shared factors underlying the association between mental disorders and cardiovascular diseases.

Several sub-studies were also started like:

(1)AngioLaus – a case control study in which 500 participants underwent detailed cardiovascular pheno-typing including intima-media thickness in the carotid arteries, pulse-wave velocity, central arterial pressure and endothelial function before and during reactive hyperaemia.

(2)HERCULES - to study the prevalence of hypertension using 24-hour ambulatory blood pressure measurement, to assess renal function using 24-hour urine collection (creatinine clearance, micro-albuminuria) and to expand understanding of genetic variants associated with hypertension and renal function within the CoLaus study.

(3)OsteoLaus - to compare risk models to discriminate osteoporotic fractures, to better understand the association of osteoporosis, cardiovascular diseases & mental health and to determine the genetic determinants of osteoporosis.

(4)HypnoLaus - to determine the prevalence of sleep disorders in the general population, to assess the genetic basis of sleep and its disorders and for better characterization of a potential link between cardiovascular disease risk factors and mental health.

Each subject had reversible anonymised unique identification barcode and samples in the bio-bank were identified with barcodes. The data are stored in an Oracle database updated with Epi-data software. A scientific advisory board consisting of external scientists evaluate the progression of the project. The participant motivation is being sustained with regular newsletters and interactive website. The project had received funding from GlaxoSmithKline initially and is currently supported by the Swiss National Science Foundation. There have been over 100 publications with more than 3000 citations so far from the project.

